# Commentary: Advancement of Knowledge of *Brucella* Over the Past 50 Years

**DOI:** 10.3389/fvets.2015.00027

**Published:** 2015-08-31

**Authors:** Giovanni Di Guardo, Sandro Mazzariol

**Affiliations:** ^1^Faculty of Veterinary Medicine, University of Teramo, Teramo, Italy; ^2^Department of Comparative Biomedicine and Food Science, University of Padova, Legnaro, Italy

**Keywords:** *Brucella ceti*, cellular prion protein, persistent organic pollutants, dioxin, dioxin-like substances, striped dolphin, *Stenella coeruleoalba*

*Brucella ceti*, a cetacean and zoonotic pathogen displaying a marked neurotropism in striped dolphins (*Stenella coeruleoalba*), appears to be the ancestor and a close relative of *B. abortus*, infecting cattle, as well as of *B. melitensis*, infecting sheep and goat (1).

In addition to the elegant mechanisms and strategies utilized by *Brucella* genus members for entering and surviving into host cells, which are clearly explained in the aforementioned review article (2), the interaction between bacterial Hsp60 – a member of the GroEL family of chaperonins – and cellular prion protein (PrP^C^) has been reported to play a crucial role in *B. abortus* infection of murine, bone marrow-derived macrophages, with no evidence of microbial colonization and replication in cells from PrP^C^-deficient mice (3, 4).

As “top predators,” cetaceans may accumulate within their tissues high concentrations of immunotoxic and neurotoxic “persistent organic pollutants” (POPs) like dioxins and dioxin-like compounds (DLCs) (5, 6), which have been demonstrated to exert pro-apoptotic activity on human neuronal cells (7). As a consequence, following dioxin-induced apoptosis, PrP^C^ overexpression is likely to occur on host cell membrane, given the anti-apoptotic role of PrP^C^, which is consistently expressed on brain cells (8).

Based upon the aforementioned model, the heavy (cerebral and extra-neural) tissue loads of lipophylic dioxins and DLCs commonly detected in stranded dolphins (5, 6) could elicit PrP^C^ overexpression on behalf of brain cells, resulting at its turn in an amplified colonization and replication activity of *B. ceti* inside these cells. The recently characterized expression patterns of PrP^C^ within the cerebral parenchyma as well as in secondary lymphoid tissues from wild dolphins (9), which resemble those previously reported in terrestrial mammals (8), provide additional support for such hypothesis, thereby making biologically plausible the occurrence of “neurobrucellosis,” a neurological disease condition frequently observed in *B. ceti-*affected striped dolphins (1). Indeed, high levels of dioxins and DLCs have been recently found in tissues from *B. ceti* meningoencephalitis-affected striped dolphins beached along the Ionian Sea coast of Italy (Antonio Petrella and Pasquale Troiano, personal communication).

In this respect, it should be also emphasized that significant differences have been recently reported in the endo-cerebral expression levels of 5-lipoxygenase (5-LOX), a neurodegeneration biomarker (10), between infectious encephalitis/meningoencephalitis-affected and -unaffected striped dolphins, with the most consistent intensity of expression of the aforementioned enzyme having been detected in a *B. ceti-*infected individual (11). This appears to be a remarkable finding, provided that such an extensive neuroinflammatory and neurodegenerative lesions’ pattern could have acted synergistically with the high tissue concentrations of dioxins and DLCs found in this dolphin (Antonio Petrella and Pasquale Troiano, personal communication), thereby leading to PrP^C^ overexpression, with subsequent putative increase of *B. ceti* colonization and replication inside brain cells.

In conclusion, based upon the strong impact exerted by *B. ceti* infection on the health and conservation *status* of free-ranging cetaceans all across the globe, associated with the proven zoonotic capability of this microorganism (1, 5, 12), the study of the infection’s neuropathogenesis, including the strategies adopted by *B. ceti* in crossing the blood–brain barrier from striped dolphins, would deserve adequate attention. Particular emphasis should be additionally placed on the study of the infection’s epidemiology and of the evolutionary genetics of *B. ceti*, also in comparison with all the other known *Brucella* genus members, as well as on the study of host–pathogen interaction dynamics, with special reference to the “host related” (PrP^C^ expression levels and patterns) and to the “environment related” (POPs, dioxins, DLCs, etc.) drivers and modulators of such complex interplay.

## Conflict of Interest Statement

The Review Editor Antonio Frangipane Di Regalbono declares that, despite being affiliated to the same institution as author Sandro Mazzariol, the review process was handled objectively and no conflict of interest exists. The authors declare that the research was conducted in the absence of any commercial or financial relationships that could be construed as a potential conflict of interest.
